# Effect of a Smart Clothes–Assisted Care System for Persons Living With Dementia on Family Caregivers: Longitudinal Nonblinded Quasi-Experimental Study

**DOI:** 10.2196/66783

**Published:** 2025-12-01

**Authors:** Ya-Li Sung, Huei-Ling Huang, Min-Chi Chen, Chung-Chih Lin, Wen-Chuin Hsu, Yea-Ing L Shyu

**Affiliations:** 1 Department of Nursing, National Chi Nan University, Nantou, Taiwan; 2 Department of Gerontology and Health Care Management, Chang Gung University of Science and Technology, Taoyuan, Taiwan; 3 Department of Public Health and Biostatistics Consulting Center, Chang Gung University, Taoyuan, Taiwan; 4 Division of Movement Disorders, Department of Neurology, Chang Gung Memorial Hospital, Taoyuan, Taiwan; 5 Department of Computer Science and Information Engineering, Chang Gung University, Taoyuan, Taiwan; 6 Dementia Center, Department of Neurology, Taoyuan Chang Gung Memorial Hospital, Taoyuan, Taiwan; 7 School of Nursing, Chang Gung University, 259 Wenhua 1st Road, Guishan District, Taoyuan, 33302, Taiwan, +886-3-2118800 ext. 5275; 8 Healthy Aging Research Center, Chang Gung University, Taoyuan, Taiwan; 9 Department of Nursing, Kaohsiung Chang Gung Memorial Hospital, Kaohsiung, Taiwan

**Keywords:** family caregivers, persons living with dementia, smart clothes–assisted care, smart technology, caregiver preparedness

## Abstract

**Background:**

Caregiving preparedness (perceived confidence in caregiving abilities) and caregiving balance (perceived confidence in finding equilibrium among overlapping demands of caregiving and personal needs) can reduce caregiver burden and depression and improve health-related quality of life. Smart clothes technology for monitoring persons living with dementia may help reduce caregiver burden and improve quality of care, but empirical evidence remains limited.

**Objective:**

This study aims to examine the preliminary effects of a smart clothes–assisted care system that monitors the daily activity of persons living with dementia on outcomes for the family caregivers.

**Methods:**

This nonrandomized, quasi-experimental study recruited dyads of family caregivers and persons living with dementia by convenience sampling from dementia care centers in northern Taiwan. A total of 60 dyads agreed to participate in the 6-month study. Persons living with dementia received either usual care (n=30, 50% control group) or usual care in addition to smart clothes–assisted care (n=30, 50% intervention group), which required wearing a smart clothes vest 24 hours per day. The nurse-led intervention was conducted in the homes of the persons living with dementia from August 2020 to November 2023. Sensors in the smart clothes vest and home monitored the activity of persons living with dementia, which was transmitted via a smartphone app to a nurse who provided caregivers with real-time feedback and individualized care plans. Caregivers completed the self-report–structured questionnaires for outcomes of caregiving preparedness, balance, depressive symptoms, and health-related quality of life at baseline and at 2, 4, and 6 months. Effects of the intervention were assessed by comparing scores and changes in scores between the intervention and control groups.

**Results:**

The mean age of the 60 caregivers was 59.32 (SD 11.46) years; most were female, married, college-educated, and co-residing with their relative. The mean age of the 60 persons living with dementia was 79.95 (SD 7.05) years; most were female, widowed, and diagnosed with Alzheimer disease; 53.5% (32/60) had a Clinical Dementia Rating of 1.0. Compared with controls, caregiving balance was significantly higher for the intervention group at 2, 4, and 6 months (*P=.*04, *P*=.01, and *P*<.001, respectively). In addition, scores for preparedness increased significantly for caregivers in the intervention group at 4 and 6 months (*P*<.05). Within-group changes from baseline for balance or mental health scores were not significant at any time point for the intervention group. However, scores for balance and mental health for the control group decreased significantly from baseline at 6 months (*P*=.01) and at 2 months (*P*=.02), respectively.

**Conclusions:**

Smart clothes–assisted care enhanced caregiver preparedness and helped prevent declines in caregiving balance and mental health. These findings provide preliminary evidence supporting the integration of wearable technology into family caregiver support interventions for persons living with dementia.

## Introduction

### Background

More than 50 million individuals across the globe are grappling with dementia. It is projected that this figure will double every 2 decades, surging to 82 million by 2030 and 152 million by 2050 [1]. In Taiwan, 7.98% of the population aged 65 years has been diagnosed with dementia, and this number is anticipated to rise from 300,000 in 2020 to 460,000 by 2041, and further to 800,000 by 2061 [2]. More than 90% of individuals diagnosed with dementia reside within their local communities, where they receive care and support primarily from family members. Thus, family members play a pivotal role in providing care to those living with dementia [3].

Family caregivers of persons living with dementia often need to balance the competing needs of the care recipient, other family members, and their own personal well-being. This notion of balance refers not to physical stability, but to the ability to manage and find equilibrium among overlapping demands, which reflects the degree to which caregivers perceive themselves as successfully maintaining this equilibrium [4]. Evidence suggests that caregivers who achieve such a balance report fewer depressive symptoms and less role strain [4-6]. Therefore, interventions that enhance caregivers’ ability to find balance across multiple domains of responsibility may contribute to better mental health and overall quality of life.

Caregiving demands encompass responsibilities related to work, household chores, and caring for other family members. However, the ability of the family caregivers to maintain their own personal well-being is difficult. Family caregivers who are able to manage the complexity of caregiving tasks and understand the needs of the care recipient are more likely to exhibit effective balance in their caregiving trajectory [6,7]. These demands influence not only balance, but also caregiving preparedness, a key outcome in dementia care research. Preparedness refers to the caregiver’s perceived readiness to provide physical assistance and emotional support and to cope with the stress of caregiving [8,9], which can be assessed using the Preparedness for Caregiving Scale. This scale evaluates one’s perceived ability to meet daily caregiving demands, provide emotional support, and handle caregiving-related challenges. Higher preparedness has been shown to predict lower caregiver burden, greater confidence, and better quality of life, making it a clinically meaningful outcome for evaluating interventions that aim to strengthen a participant’s capacity to provide family caregiving [10].

Smart clothes–assisted care has been demonstrated to help facilitate balancing caregiver responsibilities and meeting personal demands such as employment, housework, and sleep [11]. Smart clothing or smart electroclothing systems have been deployed to enhance health monitoring, optimize exercise regimens, and track physical fitness and social interactions [12,13] by enabling continuous monitoring of health metrics through sensor-based systems [14] that include abnormal activity levels, potentially hazardous postural alterations, precarious locations, and emergency situations. Advances in electronic components have made wearing smart clothing on a 24-hour basis more comfortable for remote long-term health monitoring [15]. Data collected from smart clothing are now used for illness prevention, improving compliance with rehabilitation protocols, and promoting overall well-being [13,16,17]. However, while these technologies collect biomedical or activity data and provide alerts in hazardous situations, they have made only modest contributions to reducing caregiver burden, and there is limited empirical evidence of their effectiveness in family caregiving contexts.

A feasibility study by Hou et al [11] examined whether family caregivers of older adults recovering from hip-fracture surgery and persons living with dementia would benefit from care recipients wearing a health-monitoring smart vest [18]. When home sensors and sensors in the vest detected any anomaly, alerts were sent to home care nurses via an app, who then notified caregivers. The findings demonstrated that the smart clothes–assisted care significantly reduced symptoms of depression for family caregivers, facilitated timely interventions for persons living with dementia, and helped caregivers balance work and caregiving [11]. Smart clothes–assisted care was also used during the COVID-19 pandemic with similar positive impacts on both family caregivers and persons living with dementia [19].

### Objectives

This study aimed to examine preliminary caregiver outcomes from the third year of a 3-year clinical trial of a smart clothes–assisted care system. Our design differed from previous smart clothes models because it integrated support from professional home care nurses who continuously monitored the activity of persons living with dementia as a key component of the smart clothes–assisted care intervention. Activity monitoring with sensors placed in a smart clothes vest and the home environment was designed not only to assist with the care of persons living with dementia but also to provide family caregivers with around-the-clock guidance and caregiving support from nurses. This strategy was used to improve not only the health and safety of persons living with dementia but also family caregivers’ preparedness, balance, and health-related quality of life (HRQoL).

Dyads of persons living with dementia and their family caregivers were recruited to participate in the 6-month study. Family caregivers who agreed to allow their relative to wear a smart vest 24 hours per day over the 6-month study period for the smart clothes–assisted care (intervention group) were compared with caregivers whose relative received only usual support and care (control group). We hypothesized that significantly better longitudinal improvements would be seen for measures of caregiver preparedness, balance between competing needs, symptoms of depression, and HRQoL for the intervention group compared with controls. Secondary outcomes of activities of daily living (ADLs), cognitive function, and behavioral problems were examined for the persons living with dementia. We hypothesized that these measures would be better among those who wore the smart vest compared with those who received only usual care. Positive outcomes of our study could serve as a reference for smart clothes–assisted care as a means of reducing caregiving burden for family caregivers of persons living with dementia.

## Methods

### Design

A quasi-experimental design with an intervention group and a control group, but without random assignment, was adopted. During the development of the system, we found that some homes of persons living with dementia were incompatible with the required environmental modifications to the home in order to install monitoring sensors, and some family caregivers were uncomfortable with 24-hour home monitoring [20]. Therefore, a quasi-experimental approach was used to ensure both feasibility and acceptability for all participants. Persons living with dementia in the control group of dyads received usual care. Persons living with dementia in the intervention group of dyads received usual care in addition to the smart clothes–assisted care. The study was conducted from August 1, 2020, to November 15, 2023 (ClinicalTrials.gov NCT05063045). This study was conducted and reported in accordance with the TREND (Transparent Reporting of Evaluations with Nonrandomized Designs) guidelines (Checklist 1) [21].

### Participants and Setting

A convenience sample of dyads of family caregivers and persons living with dementia was recruited from dementia clinics in a medical center in northern Taiwan. Neurologists at dementia clinics described the research study to family caregivers during regular appointments with persons living with dementia. Names of interested family caregivers were provided to research nurses, who contacted individuals over the phone to arrange a time convenient to the family caregiver to meet with the researcher at the clinic for a more detailed description of the design and purpose of the study. Family caregivers who continued to express interest in the study and the person living with dementia were eligible to participate if they met the following inclusion criteria: (1) the family caregiver was aged ≥20 years and was the person responsible for direct care or the supervision of the care received by the person living with dementia, and (2) the person living with dementia was aged >60 years, had received a diagnosis of dementia from a neurologist, and was residing full-time with either family members or the family caregiver. Caregivers were excluded if the person living with dementia had been diagnosed with a psychiatric disorder, was terminally ill, or was residing part-time in a caregiving facility.

Sample size was estimated with a generalized estimating equations approach using R software (version 4.3.0; R Foundation for Statistical Computing), including the *longpower* package [11,22] for longitudinal studies. Based on a preliminary study by Shyu [7], we used an effect size of 0.25 with comparisons between 2 groups, 4 repeated observations within each participant, and an attrition rate of 40% to reach a power of 0.8. The minimum sample size was estimated to be 36. However, to account for potential attrition and incomplete data, 60 dyads were set as the target enrollment.

### Smart Clothes–Assisted Care Intervention

The smart clothes–assisted care intervention was designed to reduce caregiver burden by continuously monitoring the daily home activities of persons living with dementia, thereby reducing the time caregivers needed to spend on direct supervision. In addition to the usual care received by both groups of persons living with dementia, as described in the Usual Care section, persons living with dementia in the intervention group also received smart clothes–assisted care by wearing a smart vest 24 hours per day over the 6-month study period. Family caregivers in the intervention group received feedback from professional home care nurses who continuously monitored the sensor data transmitted to them through a mobile app. The goal of the intervention was for nurses to provide caregivers with individualized strategies that would help reduce their time needed to supervise the activities of their relative by increasing a caregiver’s ability to better predict behaviors and the care needs of the person living with dementia. This support was expected to improve caregivers’ preparedness and balance by easing competing demands, reducing role strain, and lowering depressive symptoms, which has been demonstrated to improve the process of caregiving for family caregivers of persons living with dementia [6].

The smart vest used in this study was made of washable electroconductive fabric with coin-sized sensors sewn into the garment to prevent accidental removal [23]. The vest opened in the front for ease of use, and when buckled closed, the circuit was completed, and data were transmitted via a smartphone app to a secure server in the home nursing center. Additional sensors were placed in the living areas, bedrooms, and near the front door to capture contextual information about mobility and home safety. The data were collected from the smart clothes system and included continuous recording of steps, nocturnal activity (getting up at night), time spent in specific areas of the home (eg, bathroom), and periods of inactivity.

Baseline data were established during the first week for walking patterns and sleep schedules, with thresholds calculated as the weekly mean (SD). These thresholds were updated weekly to reflect individualized patterns, including adjustments for mobility aids. When data fell outside the established thresholds, alerts were automatically generated and transmitted to the home care nurse. These activities included bathroom stays of >15 minutes, daytime inactivity of >2 hours, excessive nocturnal awakenings, or exiting the home. Home care nurses reviewed these data daily, using deviations from the established thresholds to identify potential health or safety concerns. Caregivers were reassured that nurses would notify them immediately when deviations in activity were flagged, which was done either by phone or via the mobile phone–based LINE app (LY Corp) on the caregiver’s smartphone.

Once the intervention began, nurses shared any health or safety concerns immediately with the caregivers over the phone and suggested changes in caregiving strategies to reduce alerts. It was hoped that this design would not only relieve caregivers of the need to maintain constant vigilance of the person living with dementia but would also provide support for the caregiver by providing opportunities for sharing any caregiving challenges they were having with the home care nurse.

When a sensor in the smart vest worn by a person living with dementia triggered an alert, the home care nurse promptly contacted the family caregiver directly by phone or on the mobile phone–based LINE app, described the situation, and provided appropriate health information or a consultation. These were followed by structured safety checks every 3 days, which included individualized caregiving strategies tailored to reduce the risk of repeat alerts generated by the person living with dementia and enhance their safety. The most common alerts per day were related to exiting the home, excessive nocturnal awakening, not wearing the sensor, and low activity level. Over the 6-month intervention, the caregiving strategies provided by the nurses included instructions and educational material on how to approach the challenges of managing nocturnal restlessness, agitation, fall prevention, and behavioral problems, and how to safely increase daily activities for the person living with dementia.

### Usual Care

Usual care was received by all persons living with dementia in the 2 groups, which was provided during visits to the neurology clinic or dementia center. Care included medical evaluations, health education, case management services, and any needed social services. Case management services and consultations were provided either in person at community dementia integrated care centers or by phone. Services include caregiver training in cognitive activities for persons living with dementia and support programs for family caregivers, which are available to caregivers at dementia community service sites throughout Taiwan [24].

### Primary Outcomes for Family Caregivers

#### Caregiving Preparedness

We used the Preparedness for Caregiving Scale [9] to assess how well-prepared caregivers believed they were for caregiving. The 8-item self-report instrument assesses 7 domains of caregiving, including the ability to provide physical support, emotional support, and dealing with the stress of caregiving, and an eighth item, which asks the caregiver to give an overall rating of how well-prepared they are to be a person living with dementia [8,9]. Each item is rated on a 5-point Likert scale from 0=not at all prepared to 4=very well prepared. Total scores range from 0 to 32; the higher the score, the more prepared the caregiver feels they are for caregiving. In studies on family caregivers of frail elders in the United States, the Cronbach α ranged from 0.86 to 0.92 [9,25,26]. For this study, we assessed perceived caregiving preparedness with a Chinese version of the scale, which was demonstrated to be reliable for family caregivers of persons living with dementia in Taiwan [27]. The Cronbach α for the Chinese version of the scale was 0.92. Cronbach α for this study ranged from 0.940 to 0.945 at different time points.

#### Caregiving Balance

The degree of balance between and within competing needs of caregiving and family life was measured with the 17-item Caregiving Process of Finding a Balance Point scale [28]. Each item of the scale is a competing need, which is scored based on how well the caregiver believes they are able to balance caregiving and family life. Responses to each item are scored on a 3-point Likert scale: 1=“not able to handle either of the two”; 2=“able to handle both, but not well”; or 3=“usually able to handle both.” The overall score for balance is calculated by averaging the “balance ratings” for the items that are identified as competing needs. The Cronbach α in this study ranged from 0.72 to 0.77 at different time points.

#### Depressive Symptoms

Depressive symptoms were assessed with the Chinese version of the 20-item Center for Epidemiologic Studies–Depression Scale (CES-D) [29,30]. Each item is a symptom associated with depression. Items are scored based on the number of days during the previous week the participant experienced the symptom: 0=“<1 day,” 1=“11‐2 days,” 2=“3‐4 days,” or 3=“5‐7 days.” Total scores range from 0 to 60, with higher scores indicating a higher risk of depression. In this study, the Cronbach α for the CES-D ranged from 0.91 to 0.94 at different time points.

#### HRQoL Outcomes

Outcomes of HRQoL were assessed with the Taiwan version of the Medical Outcomes Short Form 36-Health Survey [31]. We used the Physical Component Summary (PCS), comprised of 4 subscales (physical functioning, role limitations due to physical problems, bodily pain, and general health), the Mental Component Summary (MCS), comprised of 4 subscales (vitality, social functioning, role limitations due to emotional problems, and mental health), and included the mental health subscale score of the MCS. Total summary scores range from 0 to 100, with higher scores indicating better physical and mental functioning. Standardized scores for the PCS, MCS, and the mental health subscale of the MCS were calculated using Taiwanese norms established by Tseng et al [32], with scores ≥50 considered to be a good outcome. The Cronbach α was ≥0.80, and in this study, the Cronbach α at different time points ranged from 0.91 to 0.93.

### Secondary Outcomes for Persons Living With Dementia

We selected measures for daily functioning, cognition, and behavioral problems as secondary outcomes for persons living with dementia. Outcome variables were selected to determine if continuous monitoring of activities and feedback and support provided to caregivers by the professional home care nurses would benefit the persons living with dementia in the intervention group compared with the control group, whose caregivers and persons living with dementia only received support from case management services, dementia care centers, and community services.

ADLs, such as eating, toileting, walking, climbing stairs, dressing, as well as bowel and bladder control, were assessed using the 10-item Chinese Barthel Index [33]; higher scores indicate a greater level of independence. Cognitive functioning was measured with the Taiwan version of the 11-item MMSE, which assesses orientation to time, verbal recall, language, and visual construction [34]. Total MMSE scores range from 0 to 30, with scores ≥25 indicating normal cognition, 21‐24 points indicating mild dementia, and 10‐20 points indicating moderate dementia. Behavioral problems were assessed with the Chinese version of the Cohen-Mansfield Agitation Inventory, community form [35], which has been demonstrated to be a valid and reliable measure of agitated behaviors in community-dwelling persons living with dementia in Taiwan [36]. Each item is an agitated behavior, which is scored according to frequency over the past 2 weeks, from 1 (never performs the behavior) to 7 (performs the behaviors several times an hour). To prevent overloading the caregiver with too many questions, 12 of the 43 commonly occurring behavioral problems in the questionnaire were selected for monitoring [37,38]. Thus, internal consistency was not measured because the entire scale was not used. Total scores for the 12 items range from 12 to 84; higher scores indicate more agitated behaviors.

### Data Collection

Baseline data for demographic and clinical characteristics of the family caregivers and persons living with dementia were collected after enrollment either in the clinic or the home of the person living with dementia at a time convenient for the dyads. Data collection was conducted by a trained research assistant, who administered the questionnaires in person. The assistant asked the questions directly and recorded caregivers’ responses on paper forms. Follow-up data were collected 2, 4, and 6 months after enrollment at a time and place convenient for the participants.

### Data Analysis

Data analysis was conducted with SPSS for Windows (version 22.0; IBM Corp). Descriptive statistics for demographic and clinical characteristics included the mean and SD for continuous variables and frequencies with percentages for categorical variables. Baseline characteristics between the intervention and control groups were compared using a 2-sample, 2-tailed *t* test and a chi-square test.

Analysis of the effectiveness of the smart clothes care intervention used an intention-to-treat approach. Therefore, caregiver outcomes were included for all participants enrolled in the study according to their original assigned groups, regardless of whether they adhered to or completed the treatment or withdrew from the study. Generalized estimating equation (GEE) models were used for analysis, which account for possible correlations in repeated measures over time and allow for examination of differences at different time points [39]. In our analysis, we specified a Gaussian family with an identity link as a working model for the continuous outcomes. Importantly, inference in GEE depends on the correct specification of the mean structure, and the robust (sandwich) variance estimator provides valid standard errors even if assumptions of normality are violated. Therefore, in GEE analysis, although some continuous variables may not be normally distributed, the outcome does not need to follow a strict normal distribution for the results to be valid [40,41]. We examined whether primary outcomes of caregiving preparedness and balance, depressive symptoms, and the 3 HRQoL assessments differed for the intervention group compared with the control group. The effect of the intervention on secondary outcomes (ADLs, MMSE scores, and behavior problems of persons living with dementia) was also examined. Both primary and secondary outcomes were modeled as a function of “time,” “group,” interaction between “time” and “group,” and living arrangement, due to the significantly different distribution between groups at baseline. A *P* value <.05 was considered statistically significant.

### Ethical Considerations

The study was conducted with the approval for human subject research of the study hospital ethics committee (Chang Gung Medical Foundation; approval: 201702016B0 and 201701649B0). Participants gave informed consent to participate in the study. All information collected from participants was deidentified to ensure privacy and confidentiality. Data were stored securely and accessible only to the research team. In addition, as compensation for their time and participation, each family caregiver received a gift card valued at US $7 to a convenience store after the completion of each outcome assessment.

## Results

### Study Participants

**Figure 1.**
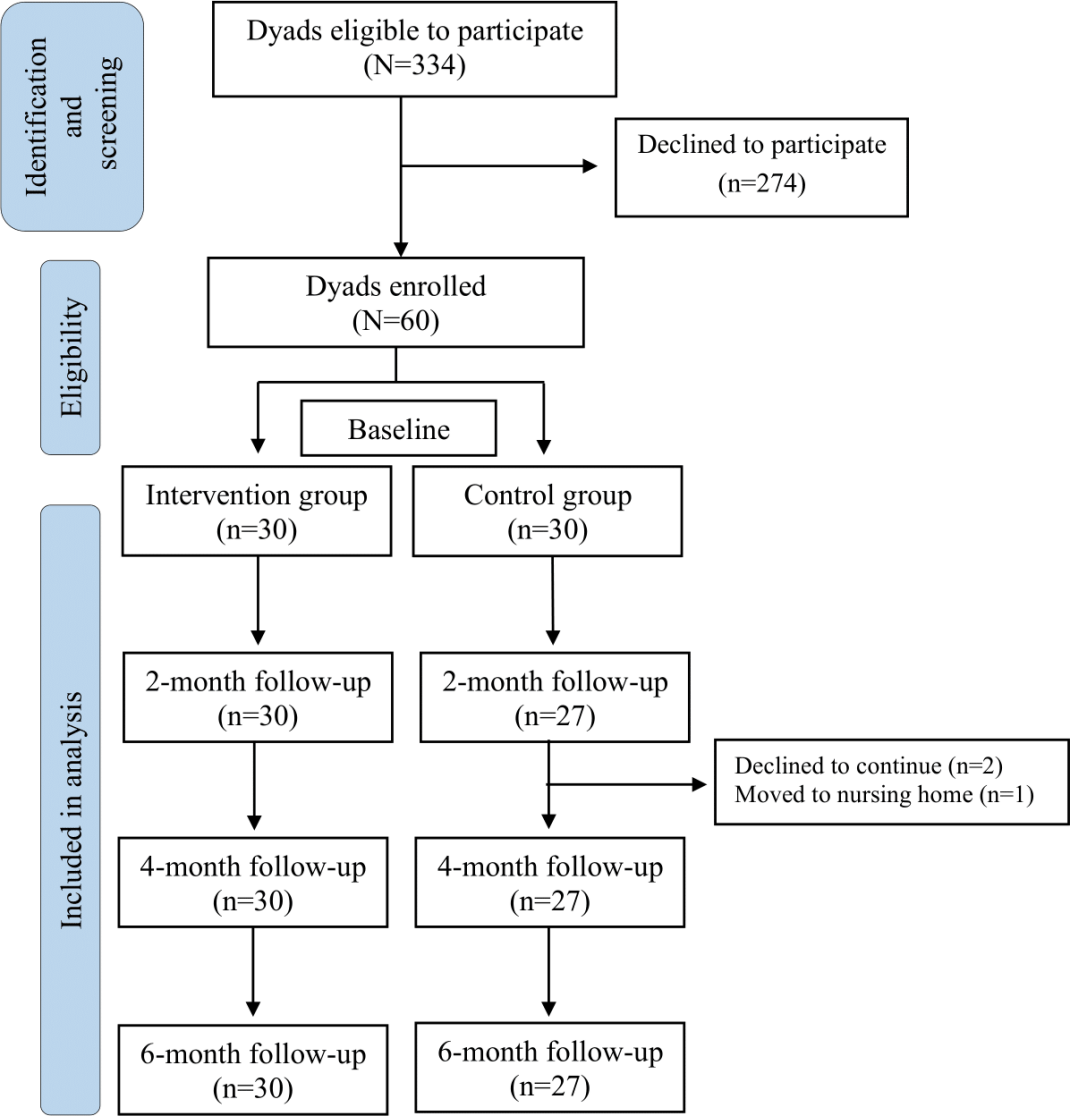
TREND (Transparent Reporting of Evaluations with Nonrandomized Designs) flow diagram.

A total of 334 dyads expressed interest and met the inclusion criteria for the study. However, 274 subsequently declined to participate due to inconvenience, not enough family manpower, or because they perceived there was no need for the smart clothes–assisted care. A total of 60 dyads provided signed informed consent to participate and were assigned to the intervention group (n=30, 50%) or control group (n=30, 50%). Three participants were lost to attrition following the 2-month follow-up; 2 had no interest in continuing, and 1 caregiver’s family member was transferred to a nursing home. Figure 1 shows the flow diagram of the study.

Characteristics of the 60 dyads and comparisons between the intervention group and control group are shown in Table 1. The mean age of all family caregivers was 59.32 (SD 11.46) years; most were female (n=38, 63%), married (n=45, 75%), and college educated (n=41, 68%). Most caregivers lived with the person with dementia (n=46, 77%) and were an adult child of the person living with dementia (n=39, 65%). Of the 30 caregivers, 26 (40%) had help from other family members, and 31 (52%) received support from community care resources. The mean age of persons living with dementia was 70.95 (SD 7.05) years. Most persons living with dementias were female (n=43, 72%), widowed (n=31, 52%), had a Clinical Dementia Rating score of 1.0 (n=32, 53%), and had been diagnosed with Alzheimer disease (n=45, 75%). The mean number of months since diagnosis of dementia was 18.5 (SD 32.47).

**Table 1.** Characteristics of all dyads of family caregivers and persons living with dementia and differences between the intervention and control groups.

Characteristic	All dyads (n=60)	Intervention group (n=30)	Control group (n=30)	*P* value
Family caregivers				
Age (y), mean (SD)	59.32 (11.46)	59.70 (10.25)	58.93 (12.73)	.79
Sex, n (%)				>.99
Female	38 (63)	19 (63)	19 (63)	
Male	22 (37)	11 (37)	11 (37)	
Marital status, n (%)				.32
Married	45 (75)	25 (83)	20 (67)	
Single	6 (10)	2 (7)	4 (13)	
Other	9 (15)	3 (10)	6 (20)	
Education, n (%)				.13
Junior high school or below	5 (8)	3 (10)	2 (7)	
High school	14 (23)	10 (33)	4 (13)	
College or above	41 (68)	17 (57)	24 (80)	
Living arrangement, n (%)				.03
With person living with dementia	46 (77)	27 (90)	19 (63)	
Separate from person living with dementia	14 (23)	3 (10)	11 (37)	
Relationship to person living with dementia, n (%)				.71
Spouse	16 (27)	9 (30)	7 (23)	
Adult child	39 (65)	18 (60)	21 (70)	
Other	5 (8)	3 (10)	2 (6.67)	
Assistance with caregiving, n (%)				.16
None	8 (13)	2 (7)	6 (20)	
Other family members	26 (43)	17 (57)	9 (30)	
Hired foreign caregiver	24 (40)	10 (33)	14 (47)	
Home attendant	2 (3)	1 (3)	1 (3)	
Caregiving resources, n (%)				.43
Yes	31 (52)	14 (47)	17 (57)	
No	29 (48)	16 (53)	13 (43)	
Outcomes at baseline, mean (SD)				
Caregiving preparedness	18.15 (5.14)	17.46 (4.62)	18.83 (6.69)	.36
Caregiving balance	2.42 (0.49)	2.52 (0.49)	2.31 (0.47)	.10
Symptoms of depression (CES-D^a^)	10.45 (8.87)	9.76 (9.09)	11.13 (8.76)	.55
HRQoL^b^, mean (SD; range 1‐100)				
PCS^c^	72.39 (7.14)	72.96 (7.16)	71.82 (7.20)	.54
MCS^d^	44.03 (8.64)	43.14 (9.18)	44.91 (8.12)	.43
MH^e^	67.53 (20.64)	66.40 (22.95)	68.6 (18.4)	.67
Persons living with dementia				
Age (y), mean (SD**)**	79.95 (7.05)	79.23 (7.20)	80.67 (6.95)	.43
Gender, n (%)				.15
Female	43 (72)	19 (63)	24 (80)	
Male	17 (28)	11 (37)	6 (20)	
Marital status, n (%)				.28
Married	26 (43)	16 (53)	10 (33)	
Widowed	31 (52)	13 (43)	18 (60)	
Other	3 (5)	1 (3)	2 (7)	
Education, n (%)				.96
Primary school	28 (47)	14 (47)	14 (47)	
Junior high school or below	5 (8)	2 (7)	3 (10)	
High school	11 (18)	6 (20)	5 (17)	
College or above	16 (27)	8 (27)	8 (27)	
Clinical dementia rating, n (%)				.55
0.5	25 (42)	14 (47)	11 (37)	
1.0	32 (53)	14 (47)	18 (60)	
2.0	3 (5)	2 (7)	1 (3)	
Type of dementia, n (%)				.30
Alzheimer disease	45 (75)	20 (67)	25 (83)	
Vascular dementia	4 (7)	3 (10)	1 (3)	
Other	11 (18.33)	7 (23.33)	4 (13.33)	
Months since diagnosis, mean (SD)	48.05 (32.47)	44.90 (32.67)	51.20 (32.52)	.45
Outcomes at baseline, mean (SD)				
MMSE^f^	19.00 (5.43)	18.73 (6.34)	19.28 (4.39)	.70
ADL^g^	89.42 (14.41)	88.83 (16.05)	90.00(12.79)	.75
Behavior problems	27.05 (6.42)	27.20 (7.21)	26.89 (5.65)	.85

a CES-D: Center for Epidemiologic Studies–Depression Scale.

b HRQoL: health-related quality of life measured with the 36-item Short Form Health Survey questionnaire.

c PCS: Physical Component Summary.

d MCS: Mental Component Summary.

e MH: Mental Health Subscale of the MCS.

f MMSE: Mini-Mental State Examination.

g ADL: activities of daily living.

### Caregiver Outcomes: Intervention Group Compared With the Control Group

GEE analysis examined if there were significant differences in outcomes for caregivers in the intervention group compared with the control group, after adjusting for the covariate of living arrangement (Table S1 in Multimedia Appendix 1). Only main effects were assessed for GEE models of depression and models of PCS and MCS for HRQoL, with no group differences observed (*P*=.23, .64, and .81, respectively). For time effects, when compared with baseline, there was a significant improvement in PCS scores at 4 months (ß=1.339, 95% CI 0.300-2.378; *P*=.01)

When interaction effects of group x time were examined, compared with the control group, the intervention group had significant improvements from baseline in scores for caregiving preparedness and caregiving balance at 6 months (ß=2.594, 95% CI 0.488-4.700; *P*=.01 and ß=0.181, 95% CI 0.001-0.361; *P*=.049, respectively) and scores on the mental health subscale at 2 months (ß=4.535, 95% CI 0.048-9.022; *P*=.048).

### Caregiver Outcomes: Between and Within Groups at Each Time Point

GEE analysis compared mean scores between and within the intervention and control groups for the 3 outcome variables that differed significantly for the interaction of group x time over 6 months, preparedness, balance, and mental health (Table S2 in Multimedia Appendix 2). Baseline scores did not differ significantly between groups for any outcomes. However, there was a statistically significant higher score in balance for the intervention group compared with the control group at 2 months (difference 0.26 points, 95% CI 0.011-0.506; *P*=.04), 4 months (difference 0.32 points, 95% CI 0.078-0.575; *P*=.01), and 6 months (difference 0.39 points, 95% CI 0.176-0.602; *P*<.001).

**Figure 2.**
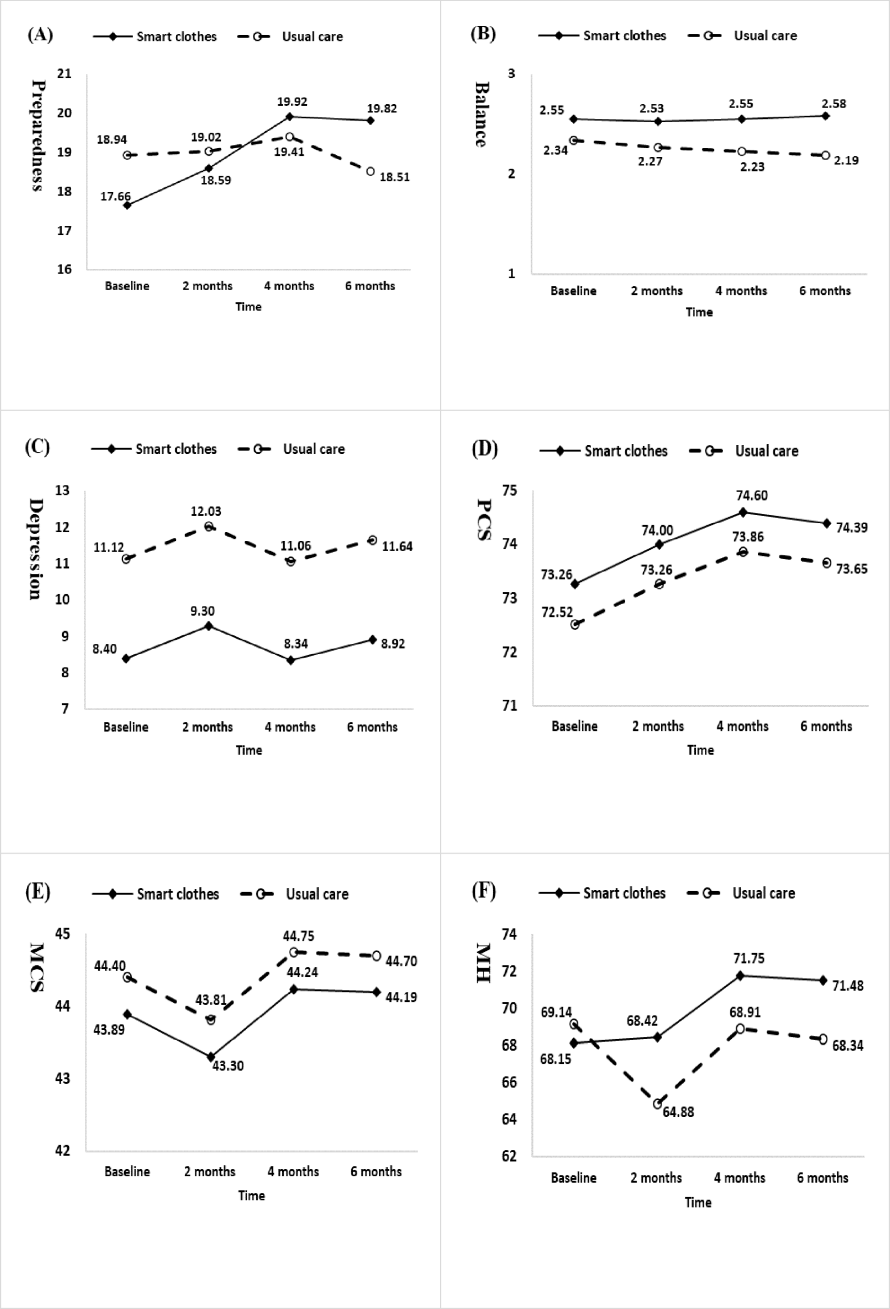
Differences in estimated mean scores for caregiver outcomes between the intervention group (smart clothes) and the control group (usual care). (A) Preparedness, (B) balance, (C) depression, (D) Physical Component Summary (PCS), (E) Mental Component Summary (MCS), and (F) Mental Health Subscale (MH) of the MCS.

When changes from baseline in outcome scores within groups were examined, there were significant improvements in preparedness in the intervention group of 2.27 (95 % CI 0.492-4.041) points at 4 months (*P*=.01) and 2.17 (95 % CI 0.534-3.798) points at 6 months (*P*=.009). Within-group changes from baseline in outcome scores of balance or mental health were not significant at any time point for the intervention group. Changes in scores from baseline within the control group demonstrated no significant improvements for any of the 3 outcome variables. By contrast, outcome scores for balance decreased significantly from baseline by 0.15 (95% CI –0.271 to –0.030) points for balance at 6 months (*P*=.01) and 4.26 (95% CI –7.988 to –0.549) points for mental health at 2 months (*P*=.02). Differences between and within the 2 groups for the 6 primary outcome variables over time are illustrated in Figure 2.

### Effects of the Intervention on Secondary Outcomes for Persons Living With Dementia

There were no statistically significant differences for any of the secondary outcomes for persons living with dementia in the intervention group compared with the control group at any time point. However, there were significant changes from baseline in the secondary outcomes of MMSE scores and the number of behavioral problems within both groups for persons living with dementia. There was a statistically significant change from baseline in MMSE scores at 2 months (0.573 points; *P*=.03). There was also a statistically significant decrease from baseline in the number of behavior problems at 2 months (−1.114; *P*=.02), which continued at 4 months (−1.462; *P*=.049), but not at 6 months.

## Discussion

### Principal Findings

The preliminary outcomes in this study demonstrated that family caregivers of persons living with dementia who received the smart clothes–assisted care intervention had statistically significant improvements from baseline for measures of caregiving preparedness and balance at 6 months compared with the family caregivers of persons living with dementia in the control group who received usual care. These findings partially support our hypothesis that the smart clothes–assisted care intervention would result in longitudinal improvements in caregiver preparedness and balance. However, there were no statistically significant longitudinal improvements in depression or variables of HRQoL for caregivers in the intervention group compared with the control group.

This is the first study to provide quantitative data supporting the effectiveness of smart clothes technology designed for monitoring the activity of persons living with dementia on family caregivers. Most smart care technologies that involve wearable and home-based sensor systems have focused on monitoring health parameters and rehabilitation, with limited evidence of the impact on outcomes for caregivers. We augmented the smart clothes system by integrating smart clothes monitoring with alert-specific support from professional home care nurses, which allowed us to target family caregivers.

Our findings highlight the potential benefits of our smart clothes–assisted care model in improving caregiver preparedness and balance. However, our intervention is also more demanding compared with prior smart care models, as it requires considerable time and involvement from home care nurses, which may impact the feasibility of offering this as a form of long-term care in terms of both cost and manpower. We suggest addressing this limitation in future studies through modifications that restructure the platform for the caregiver-nurse interactions such that caregivers receive real-time feedback about care as well as educational push notifications. This could enhance automation that not only further improves caregiver and patient outcomes but also reduces staff workload and increases the likelihood that smart clothes–assisted care would be sustained in routine home care settings.

Although the increase in caregiving preparedness within the intervention group at 6 months was statistically significant compared with baseline, the range of possible scores (0=“not prepared at all” to 32=“extremely prepared,” 0‐32 points) is broad. Therefore, while an improvement of 2.27 points was statistically significant, it may represent only a modest change in caregivers’ perceived preparedness and requires further evaluation to determine whether it is clinically meaningful. Similarly, the statistically significant improvements in caregiving balance from baseline observed at 2, 4, and 6 months were small and ranged from 0.26 to 0.39 (1=“not able to handle both” to 3=“usually able to handle both,” 1‐3 points), and may reflect minor perceived benefits in balancing caregiving with personal needs. These findings also warrant further validation regarding their practical importance. Future studies should examine whether such improvements translate into tangible outcomes, such as reduced caregiver burden, fewer depressive symptoms, or enhanced coping and care quality. Nonetheless, caregiving demands are persistent and cumulative for caregivers of persons living with dementia, and in the context of dementia care, even small gains in preparedness and balance have been shown to mitigate long-term strain and strengthen caregivers’ resilience over time [42].

There were no significant improvements for secondary outcomes of ADLs, MMSE, and scores, or behavioral problems for persons living with dementia in the intervention group compared with the control group. One explanation for the lack of an improvement may be explained by the relatively short 6-month follow-up period, which might not have been sufficient for measurable psychological or functional changes to emerge or due to the limited sample size, which may have reduced the statistical power to detect small to moderate effects. However, there were small but significant increases in MMSE scores at 2 months and a reduction in the number of behavior problems at 2 months and 4 months for persons living with dementia in both groups. These improvements may reflect caregiver-mediated effects. As both groups received regular follow-up contact from home care nurses, the engagement itself may have enhanced caregivers’ awareness, prompted earlier responses to behavioral changes, and encouraged more structured daily routines for persons living with dementia. Such indirect effects highlight the importance of caregiver involvement as an active component of dementia care interventions, even when technological or educational elements differ between groups.

### Comparisons With Prior Work

The benefits of significant improvements in preparedness and balance for caregivers in the intervention group in our study are consistent with a pilot study by Hou et al [11]. Reports from face-to-face interviews with 7 family caregivers of persons living with dementia indicated that the smart clothes–assisted care program facilitated preparedness and increased their work-life balance because of the guidance received from the home care nurses. Our findings expand upon the previous study [11] by providing quantitative data from a large group of family caregivers and comparing caregivers of persons living with dementia who received the smart clothes–assisted care with a control group of caregivers of persons living with dementia who received usual care. Managing the needs of care recipients and personal needs simultaneously can lead to high levels of stress for family caregivers of persons living with dementia [43]. Our findings suggest the better scores for preparedness and balance in the intervention group were the result of being better able to balance work and caregiving roles, which has been demonstrated to increase role strain [44].

There was a benefit of significant increases in mental health for the intervention group as demonstrated by the interaction effect of group x time as well as within-group changes from baseline at 2 months. Although caregivers who can balance competing needs are more likely to have better mental health [5,28,44], our finding suggests a large improvement in balance may be necessary for long-term improvements. In addition, the ability to balance competing needs has been found to mediate the association between caregiving demands and caregiver role strain and depressive symptoms for family caregivers of persons living with dementia [4].

Our study did not find an effect of the intervention on depression or HRQoL. The failure of the intervention to reduce depression contrasts with previous studies on smart clothes–assisted care [11,19]. However, as mentioned earlier, the study by Hou et al [11] included only 7 family caregivers of persons living with dementia and had no control group for comparison, and the reduction in depression reported by Sung et al [19] was the result of qualitative interview data only. In addition, despite the improvement in caregiver balance, as was seen for participants in the intervention group, the smart clothes–assisted care intervention may not have reduced caregiving demands sufficiently to have a significant effect on depression or HRQoL as measured with the CES-D or Short Form 36-Health Survey, respectively. By contrast, the lack of a reduction in depression or improvements in HRQoL is in agreement with a recent systematic review and meta-analysis of 12 studies on telehealth applications for family caregivers of persons living with dementia [45], which found no significant difference in depression or HRQoL for caregivers who received the telehealth support intervention compared with the control groups. The relatively short 6-month follow-up period as well as the limited sample size might not have been sufficient for measurable psychological or functional changes to emerge, or may have reduced the statistical power to detect small to moderate effects. Thus, an effect of smart clothes interventions on broader psychosocial outcomes such as depression and HRQoL may require a longer period of time, more support from nurses, or a larger sample to detect a measurable effect. We suggest additional quantitative smart clothes–assisted care studies of ≥1 year be conducted. In addition, conducting qualitative interviews with caregivers at the conclusion of the intervention might provide additional information on how the intervention might have a more robust impact on reducing symptoms of depression and improving HRQoL for family caregivers of persons living with dementia.

Our findings align with studies on the benefits of smart-home technologies, which not only monitor the behaviors and activities of older care recipients but also inform caregivers and health care professionals about potential risks [46]. These technologies can also assist family caregivers by improving connections between caregivers and health care professionals [47]. The functions included in the design of the smart vest used in this study were based on a previous study demonstrating that monitoring ADLs and falls not only enhances safety but also allows older community-dwelling adults to remain at home [48]. We believe that the significant improvements in caregiving preparedness and balance for caregivers in the intervention group may increase their willingness to maintain their role as a family caregiver for a person living with dementia.

Family caregivers received immediate feedback from nurses on caregiving strategies to help reduce activities by the person living with dementia that generated sensor alerts, increase their safety, and provide support for mitigating caregiving difficulties. However, our findings demonstrated no improvements in any secondary outcomes for persons living with dementia in the intervention group compared with the control group. While caregiver training that enhances self-efficacy and preparedness can reduce behavioral problems in persons living with dementia [36,49] and caregiver-delivered cognitive interventions have been reported to improve cognition [50], the focus of this smart clothes–assisted care intervention was not on professional support for the persons living with dementia but on support that would relieve caregivers of the need to maintain constant vigilance. The focus of future studies will expand the role of nurses in supporting persons living with dementia.

### Strengths and Limitations

The strength of this study lies in providing preliminary evidence that the smart clothes–assisted care model designed to address safety concerns and enhance caregiver preparedness may support family caregivers of persons living with dementia. Wearing the smart vest 24/7 enabled continuous monitoring of the person living with dementia, and the immediate transmission of alerts to the home care nurses allowed nurses to provide caregivers with prompt, individualized feedback. However, given the small effect sizes and lack of significant changes in patient secondary outcomes, the findings should be interpreted cautiously. Further research with larger samples and longer follow-up is needed to explore and validate the potential benefits of this intervention in more detail.

Despite its strengths, this study had some limitations. First, the use of a nonrandomized quasi-experimental research design reduces the strength of our findings. However, we believe including living arrangements as a covariate at baseline might minimize the impact. Second, participants were recruited by convenience sampling, and the setting was limited to northern Taiwan, which might limit the generalizability of the findings. Third, due to difficulties in recruiting families, we did not stratify participants by variables such as dementia severity, which might have provided additional insights into the differential effects of the intervention. Fourth, there was a relatively high degree of refusal and dropout, likely reflecting the demanding nature of this intervention compared with other smart-care technologies. Factors such as the added burden of adaptations needed to make the home environment compatible with the sensors and technology-related difficulties, the adherence to wearing the vest 24/7 that was required of the persons living with dementia, and concerns about privacy may also have contributed to attrition. These barriers may limit the accessibility and uptake of the intervention, and future studies are needed to address these challenges. Finally, there was incomplete data for three participants in the control group, which might have biased our findings. However, there was no statistically significant difference for any characteristic between participants with complete data and those lost to attrition.

### Conclusions

The smart clothes–assisted care intervention allowed constant in-home monitoring, alarm signals, and feedback from home care nurses. Our findings provide support for the application of smart clothes–assisted care for enhancing caregiving preparedness and balance as a means of reducing the competing demands of family caregiving for persons living with dementia and fulfilling the personal needs of caregivers. We suggest similar programs could be refined and incorporated into long-term care policies for persons living with dementia. The results of this study can serve as a reference for designing and modifying future development in smart-home technology for supporting family caregivers of persons living with dementia.

## Supplementary material

10.2196/66783 Multimedia Appendix 1 Generalized estimating equation models of family caregiver outcomes for the intervention group compared with the control group.

10.2196/66783 Multimedia Appendix 2 Generalized estimating equation analysis of differences in scores between groups and changes in scores from baseline within groups for caregiver outcomes with significant interaction effects.

10.2196/66783 Checklist 1 TREND checklist.

## Abbreviations

ADL: activities of daily living
CES-D: Center for Epidemiologic Studies–Depression Scale
GEE: generalized estimating equation
HRQoL: health-related quality of life
MCS: Mental Component Summary
MMSE: Mini-Mental State Examination
PCS: Physical Component Summary
TREND: Transparent Reporting of Evaluations with Nonrandomized Designs

## References

[1] Guerchet M, Prince M, Prina M (2020). Numbers of people with dementia worldwide: an update to the estimates in the world alzheimer report 2015. Alzheimer’s Disease International.

[2] Understanding dementia: dementia in Taiwan. Taiwan Alzheimer’s Disease Association.

[3] (2021). The Senior Citizen Condition Survey 2017—the Primary Family Caregivers Survey Report. Ministry of Health and Welfare.

[4] Liu HY, Yang CT, Wang YN (2017). Balancing competing needs mediates the association of caregiving demand with caregiver role strain and depressive symptoms of dementia caregivers: a cross-sectional study. J Adv Nurs.

[5] Shyu YIL (2002). A conceptual framework for understanding the process of family caregiving to frail elders in Taiwan. Res Nurs Health.

[6] Liu HY, Hsu WC, Shyu YIL (2021). Finding a balance in family caregiving for people with dementia: a correlational longitudinal study. J Adv Nurs.

[7] Shyu YI (2000). Development and testing of the Family Caregiving Factors Inventory (FCFI) for home health assessment in Taiwan. J Adv Nurs.

[8] Archbold PG, Stewart BJ, Greenlick MR, Harvath TA, Funk SG, Champagne MT, Tornquist EM, Copp LA (1992). Key Aspects of Eldercare.

[9] Archbold PG, Stewart BJ, Greenlick MR, Harvath T (1990). Mutuality and preparedness as predictors of caregiver role strain. Res Nurs Health.

[10] Karg N, Graessel E, Randzio O, Pendergrass A (2018). Dementia as a predictor of care-related quality of life in informal caregivers: a cross-sectional study to investigate differences in health-related outcomes between dementia and non-dementia caregivers. BMC Geriatr.

[11] Hou YJ, Zeng SY, Lin CC (2022). Smart clothes-assisted home-nursing care program for family caregivers of older persons with dementia and hip fracture: a mixed-methods study. BMC Geriatr.

[12] Muhammad Sayem AS, Hon Teay S, Shahariar H, Fink PL, Albarbar A (2020). Review on smart electro-clothing systems (SeCSs). Sensors (Basel).

[13] Porciuncula F, Roto AV, Kumar D (2018). Wearable movement sensors for rehabilitation: a focused review of technological and clinical advances. PM R.

[14] Mohammadzadeh N, Gholamzadeh M, Saeedi S, Rezayi S (2023). The application of wearable smart sensors for monitoring the vital signs of patients in epidemics: a systematic literature review. J Ambient Intell Humaniz Comput.

[15] Ahsan M, Teay SH, Sayem ASM, Albarbar A (2022). Smart clothing framework for health monitoring applications. Signals.

[16] Ajami S, Teimouri F (2015). Features and application of wearable biosensors in medical care. J Res Med Sci.

[17] Patel S, Park H, Bonato P, Chan L, Rodgers M (2012). A review of wearable sensors and systems with application in rehabilitation. J Neuroeng Rehabil.

[18] Lin CC, Yang CY, Zhou Z, Wu S (2018). Intelligent health monitoring system based on smart clothing. Int J Distrib Sens Netw.

[19] Sung YL, Huang HL, Lin CC (2022). Experiences of family caregivers of persons living with dementia with and without a smart-clothes assisted home nursing program during the heightened COVID-19 alert. BMC Geriatr.

[20] Lin CC, Yang CT, Su PL, Hsu JL, Shyu YIL, Hsu WC (2024). Implementation difficulties and solutions for a smart-clothes assisted home nursing care program for older adults with dementia or recovering from hip fracture. BMC Med Inform Decis Mak.

[21] Des Jarlais DC, Lyles C, Crepaz N, TREND Group (2004). Improving the reporting quality of nonrandomized evaluations of behavioral and public health interventions: the TREND statement. Am J Public Health.

[22] Diggle PJ, Heagerty PJ, Liang K, Zeger SL (2002). Analysis of Longitudinal Data.

[23] Wang J, Lin CC, Yu YS, Yu TC (2015). Wireless sensor-based smart-clothing platform for ECG monitoring. Comput Math Methods Med.

[24] Hsiao YH, Kuo TA, Tai CJ (2019). Integrated care for dementia in Taiwan: policy, system, and services. Int J Gerontol.

[25] Archbold PG, Stewart BJ, Miller LL (1995). The PREP system of nursing interventions: a pilot test with families caring for older members. Res Nurs Health.

[26] Hagen CM, Archbold PG, Miller LL (2023). How tailoring led to variation in care issues, dosage, and outcomes: Part 1: Secondary analysis of the PREP trial for frail older adults and family caregivers. Res Gerontol Nurs.

[27] Shyu YIL, Yang CT, Huang CC, Kuo HC, Chen ST, Hsu WC (2010). Influences of mutuality, preparedness, and balance on caregivers of patients with dementia. J Nurs Res.

[28] Liu HY, Wang YN, Huang HL (2014). Psychometric properties of the finding a balance scale for family caregivers of elders with dementia in Taiwan. Res Nurs Health.

[29] Fu CC, Lee YM, Chen JD (2003). Association between depressive symptoms and twelve-year mortality among elderly in a rural community in Taiwan. J Formos Med Assoc.

[30] Radloff LS (1977). The CES-D scale: a self-report depression scale for research in the general population. Appl Psychol Meas.

[31] Lu JF, Tseng HM, Tsai YJ (2003). Assessment of health-related quality of life in Taiwan (I): development and psychometric testing of SF-36 Taiwan version. Taiwan J Public Health.

[32] Tseng HM, Lu JF, Tsai YJ (2003). Assessment of health-related quality of life in Taiwan (II): norming and validation of SF-36 Taiwan version. Taiwan J Public Health.

[33] Leung SOC, Chan CCH, Shah S (2007). Development of a Chinese version of the Modified Barthel Index—validity and reliability. Clin Rehabil.

[34] Yip PK, Shyu YI, Liu SI, Lee JY, Chou CF, Chen RC (1992). An epidemiological survey of dementia among elderly in an urban district of Taipei. Acta Neurol Sin.

[35] Lai CKY (2002). The use of the Cohen-Mansfield Agitation Inventory in the assessment of agitation in people with dementia: applicability in Hong Kong. Hong Kong J Gerontol.

[36] Huang HL, Kuo LM, Chen YS (2013). A home-based training program improves caregivers’ skills and dementia patients’ aggressive behaviors: a randomized controlled trial. Am J Geriatr Psychiatry.

[37] Huang HL, Shyu YL, Hsu WC (2018). Agitated behaviors among elderly people with dementia living in their home in Taiwan. Clin Interv Aging.

[38] Huang SS (2022). Depression among caregivers of patients with dementia: associative factors and management approaches. World J Psychiatry.

[39] Liang KY, Zeger SL (1993). Regression analysis for correlated data. Annu Rev Public Health.

[40] Liang KY, Zeger SL (1986). Longitudinal data analysis using generalized linear models. Biometrika.

[41] Zeger SL, Liang KY, Albert PS (1988). Models for longitudinal data: a generalized estimating equation approach. Biometrics.

[42] Uhm KE, Jung H, Woo MW (2023). Influence of preparedness on caregiver burden, depression, and quality of life in caregivers of people with disabilities. Front Public Health.

[43] Wang YN, Shyu YIL, Chen MC, Yang PS (2011). Reconciling work and family caregiving among adult-child family caregivers of older people with dementia: effects on role strain and depressive symptoms. J Adv Nurs.

[44] Shyu YIL, Chen MC, Wu CC, Cheng HS (2010). Family caregivers’ needs predict functional recovery of older care recipients after hip fracture. J Adv Nurs.

[45] Söylemez BA, Özgül E, Küçükgüçlü Ö, Yener G (2023). Telehealth applications used for self-efficacy levels of family caregivers for individuals with dementia: a systematic review and meta-analysis. Geriatr Nurs.

[46] Amiribesheli M, Benmansour A, Bouchachia A (2015). A review of smart homes in healthcare. J Ambient Intell Human Comput.

[47] Adler R, Mehta R (2014). Catalyzing technology to support family caregiving. National Alliance for Caregiving.

[48] Verloo H, Kampel T, Vidal N, Pereira F (2020). Perceptions about technologies that help community-dwelling older adults remain at home: qualitative study. J Med Internet Res.

[49] Huang HL, Shyu YIL, Hsu WC, Liao YT, Huang HL, Hsieh SH (2024). Effectiveness of a health education program for people with dementia and their family caregivers: An intervention by nurse practitioners. Arch Psychiatr Nurs.

[50] Silva R, Bobrowicz-Campos E, Santos-Costa P (2023). Effectiveness of caregiver-provided individual cognitive interventions in older adults with dementia. J Alzheimers Dis Rep.

