# Supplementary material for: Effect of a Smart Clothes–Assisted Care System for Persons Living With Dementia on Family Caregivers: Longitudinal Nonblinded Quasi-Experimental Study
Source: J Med Internet Res. 2025 Dec 1;27:e66783. doi: 10.2196/66783 (PMC12670050; doi:10.2196/66783)
Supplement: Multimedia Appendix 1 [file jmir-v27-e66783-s001.docx]

**Table S1.** Generalized estimating equation models of family caregiver outcomes for the intervention group compared with the control group

|  | **Preparedness** | |  | **Balance** | |  |
| --- | --- | --- | --- | --- | --- | --- |
| Parameter | ß | 95% CI | *P* | ß | 95% CI | *P* |
| Intercept | 19.203 | 15.892, 22.514 | <.001 | 2.381 | 2.192, 2.570 | <.001 |
| Living arrangement ^a^ | -0.525 | -4.129, 3.079 | .77 | -0.077 | -0.278, 0.124 | .45 |
| Group ^b^ | -1.282 | -4.308, 1.744 | .40 | 0.208 | -0.037, 0.453 | .09 |
| Time^c^ |  |  |  |  |  |  |
| 2 months | 0.083 | -0.477, 0.643 | .77 | -0.070 | -0.150, 0.010 | .08 |
| 4 months | 0.476 | -0.287, 1.239 | .22 | -0.113 | -0.246, 0.021 | .09 |
| 6 months | -0.427 | -1.757, 0.903 | .52 | -0.151 | -0.271, -0.031 | .01 |
| Interaction ^c^, Group x Time |  |  |  |  |  |  |
| 2 months | 0.850 | -0.483, 2.184 | .21 | 0.051 | -0.084, 0.186 | .45 |
| 4 months | 1.791 | -0.143, 3.724 | .06 | 0.119 | -0.085, 0.322 | .25 |
| 6 months | 2.594 | 0.488, 4.700 | .01 | 0.181 | 0.001, 0.361 | .049 |
|  |  |  |  |  |  |  |
|  | **Depression** ^d^ | |  | **PCS (**HRQoL**)** ^d^ | |  |
|  | ß | 95% CI | *P* | ß | 95% CI | *P* |
| Intercept | 10.581 | 6.744, 14.419 | **<**.001 | 73.390 | 70.813, 75.966 | <.001 |
| Living arrangement ^a^ | 1.081 | -4.163, 6.326 | .68 | -1.726 | -5.204, 1.753 | .33 |
| Group ^b^ | -2.721 | -7.239, 1.796 | .23 | 0.736 | -2.399, 3.871 | .64 |
| Time^c^ |  |  |  |  |  |  |
| 2 months | 0.904 | -0.703, 2.455 | .11 | 0.742 | -0.087, 1.571 | .07 |
| 4 months | -0.064 | -2.520, 2.256 | .94 | 1.339 | 0.300, 2.378 | .01 |
| 6 months | 0.520 | -1.078, 2.642 | .46 | 1.124 | -0.402, 2.650 | .14 |
|  |  |  |  |  |  |  |
|  | **MCS (**HRQoL**)** ^d^ | |  | **Mental Health (**HRQoL**)** ^d^ | |  |
|  | ß | 95% CI | *P* | ß | 95% CI | *P* |
| Intercept | 44.778 | 40.759, 48.798 | <.001 | 71.540 | 62.551, 80.529 | <.001 |
| Living arrangement ^a^ | -0.741 | -5.742, 4.261 | .77 | -4.781 | -16.855, 7.293 | .43 |
| Group ^b^ | -0.509 | -4.855, 3.837 | .81 | -0.996 | -12.250, 10.258 | .86 |
| Time^c^ |  |  |  |  |  |  |
| 2 months | -0.591 | -1.724, 0.542 | .30 | -4.269 | -7.988, -0.550 | .02 |
| 4 months | 0.344 | -1.113, 1.800 | .64 | -0.234 | -4.484, 4.016 | .91 |
| 6 months | 0.297 | -0.891, 1.485 | .62 | -0.803 | -4.414, 2.807 | .66 |
| Interaction, Group^b^ x Time^c^ |  |  |  |  |  |  |
| 2 months |  |  |  | 4.535 | 0.049, 9.022 | .048 |
| 4 months |  |  |  | 3.834 | -2.836, 10.504 | .26 |
| 6 months |  |  |  | 4.137 | -1.503, 9.777 | .15 |

ß, standardized beta; CI, confidence interval; Depression, Score on the Center for Epidemiologic Studies-Depression Scale; HRQoL, Health-related quality of life; PCS, Physical Component Summary score on the SF-36; MCS, Mental Component Summary score on the SF-36; Mental Health, mental health subscale on the MCS

^a^ Reference: Not living together

^b^ Reference: Control group

^c^ Reference: Scores at baseline assessment

^d^ Interactions of Group x Time were not included because they were not statistically significant.
